# Supplementary material for: Mother–Infant Co-Sleeping and Maternally Reported Infant Breathing Distress in the UK Millennium Cohort
Source: Int J Environ Res Public Health. 2020 Apr 25;17(9):2985. doi: 10.3390/ijerph17092985 (PMC7246529; doi:10.3390/ijerph17092985)
Supplement: Supplementary file 1 [file ijerph-17-02985-s001.pdf]

Supplementary Materials

Table S1. Correlations between logistic regression predictor variables.

Figure S1. Plots of relationships of all covariates with occurrence of breathing difficulties. Lines are Lowess smooth curves.

Table S1. Matrix of correlations for variables entered in logistic regression analysis.

Variables	(1)	(2)	(3)	(4)	(5)	(6)	(7)	(8)	(9)	(10)	(11)
(1) bed co-sleep	1.000										
(2) Breast fed	0.051	1.000									
(3) Hosp. stay	−0.024	−0.017	1.000								
(4) Infant sex	−0.023	0.004	0.013	1.000							
(5) Birthweight	−0.023	0.028	0.145	−0.106	1.000						
(6) Income score	−0.104	0.249	−0.001	0.003	0.082	1.000					
(7) Mo. Birth yr.	−0.041	0.226	0.007	0.010	0.061	0.348	1.000				
(8) Inf. n. illness	0.009	0.015	−0.021	−0.053	−0.022	0.035	−0.031	1.000			
(9) Mother educ.	−0.013	−0.238	0.022	−0.010	−0.038	−0.343	−0.259	−0.015	1.000		
(10) Dad absent	−0.093	0.174	0.003	0.007	0.063	0.321	0.290	−0.001	−0.128	1.000	
(11) Singleton	−0.021	−0.020	−0.078	0.020	−0.193	−0.015	0.050	−0.012	−0.013	0.020	1.000

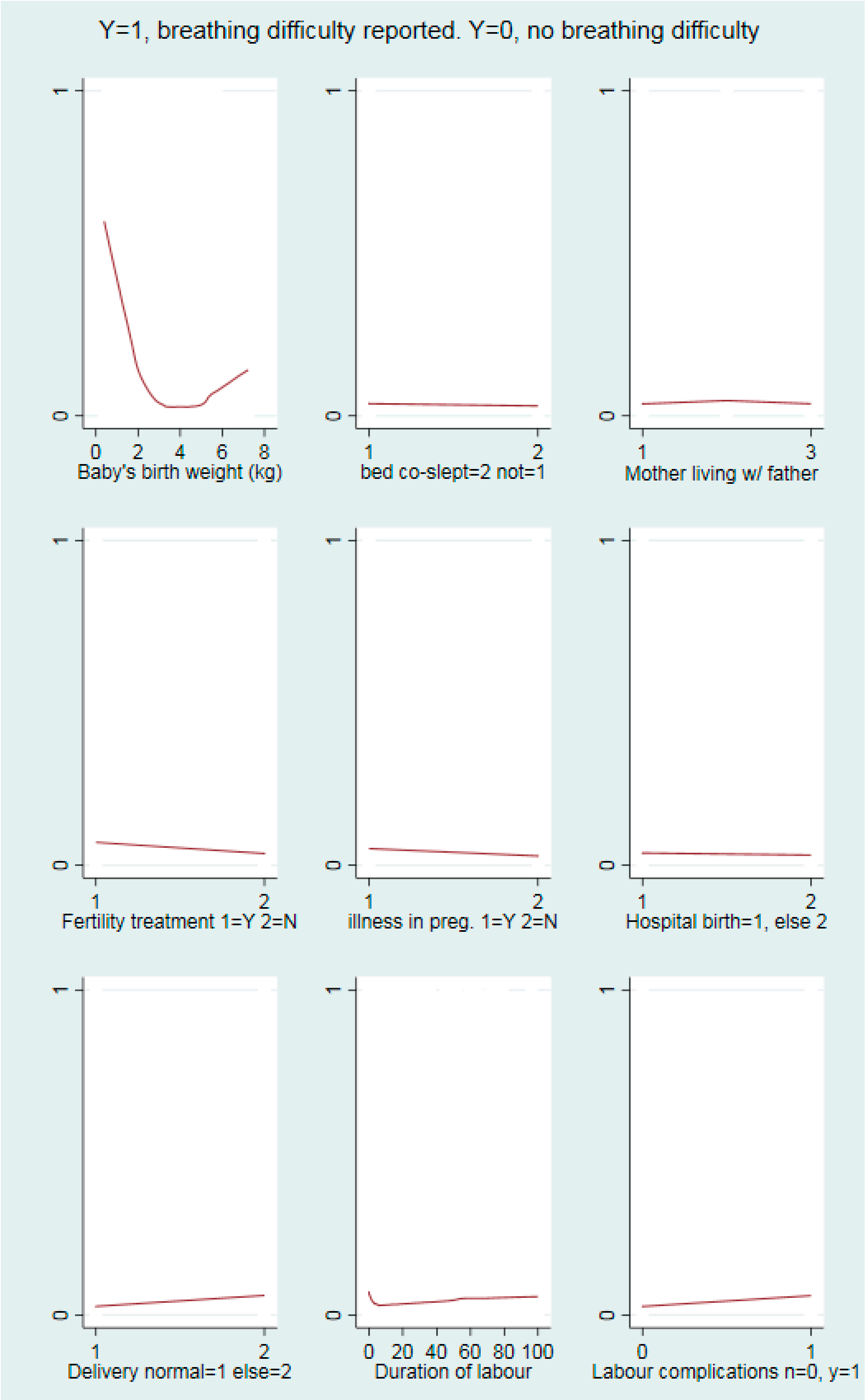

Y=1, breathing difficulty reported. Y=0, no breathing difficulty
1
0
0 2 4 6 8
Baby's birth weight (kg)
1 2
bed co-slept=2 not=1
1 3
Mother living w/ father
1 2
Fertility treatment 1=Y 2=N
1 2
illness in preg. 1=Y 2=N
1 2
Hospital birth=1, else 2
1 2
Delivery normal=1 else=2
0 20 40 60 80 100
Duration of labour
0 1
Labour complications n=0, y=1

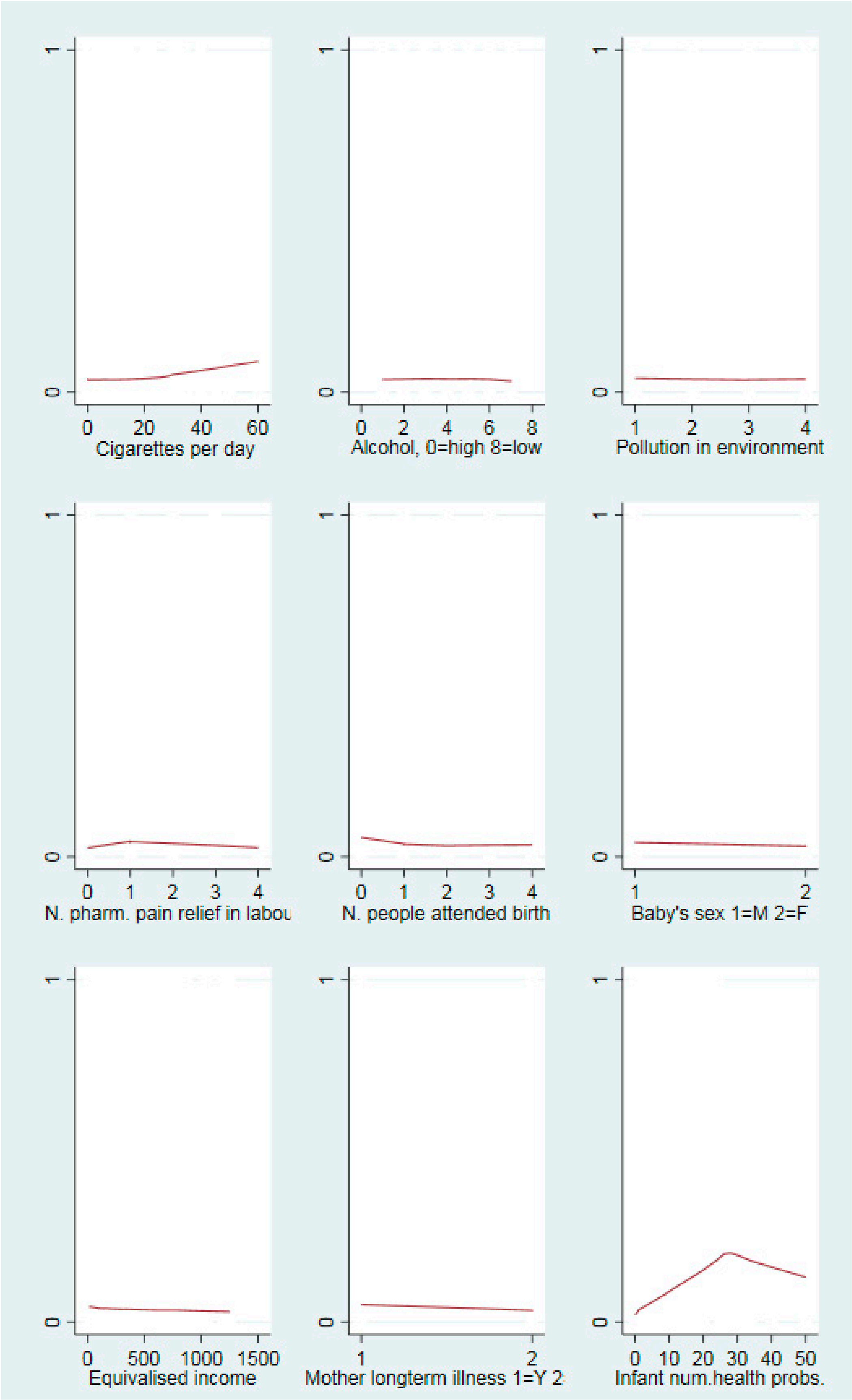

1
0
0 20 40 60
Cigarettes per day
0 2 4 6 8
Alcohol, 0=high 8=low
1 2 3 4
Pollution in environment
0 1 2 3 4
N. pharm. pain relief in labou
0 1 2 3 4
N. people attended birth
1 2
Baby's sex 1=M 2=F
0 500 1000 1500
Equivalised income
1 2
Mother longterm illness 1=Y 2
0 10 20 30 40 50
Infant num.health probs.

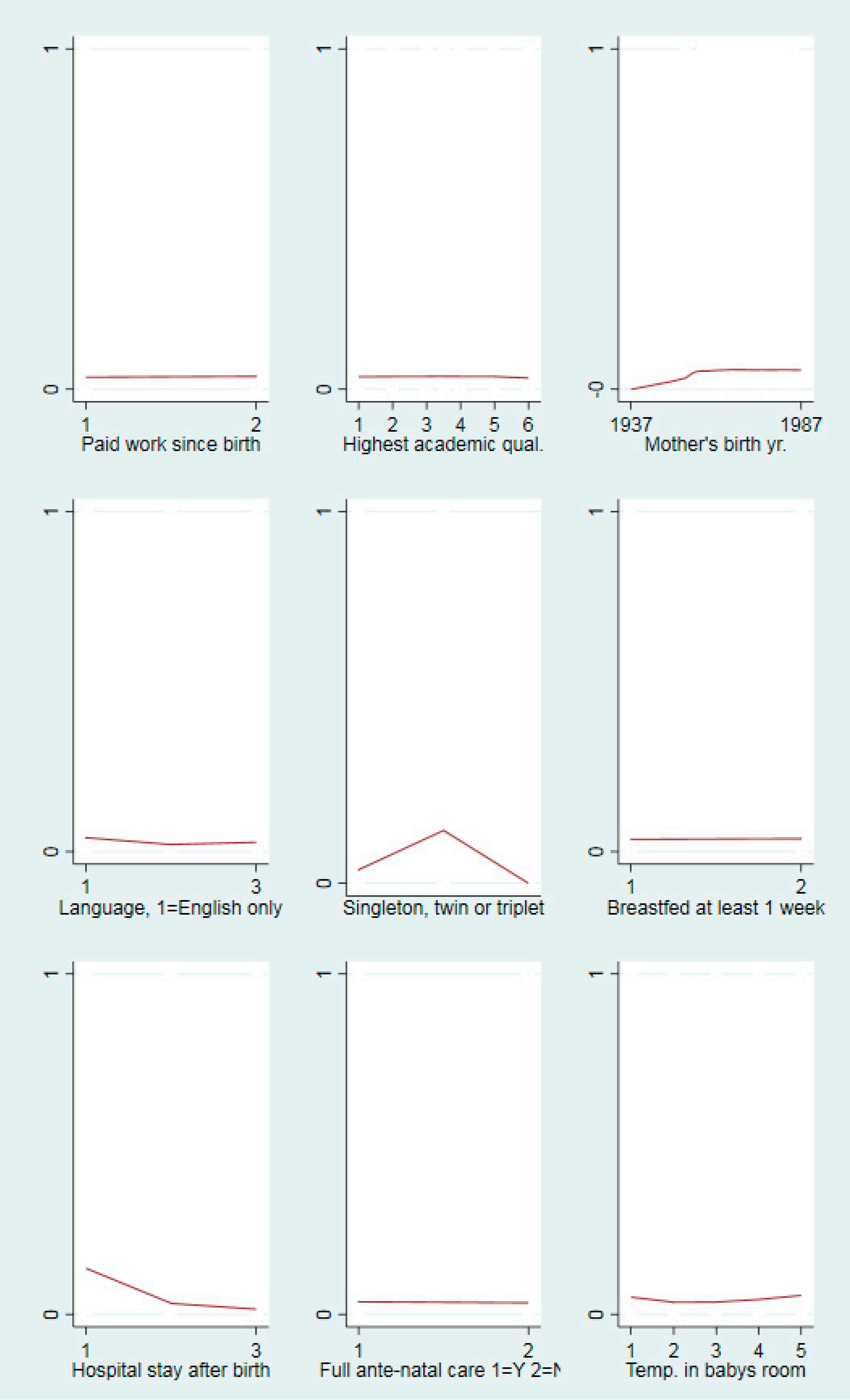

Paid work since birth
Highest academic qual.
Mother's birth yr.
Language, 1=English only
Singleton, twin or triplet
Breastfed at least 1 week
Hospital stay after birth
Full ante-natal care 1=Y 2=N
Temp. in babys room

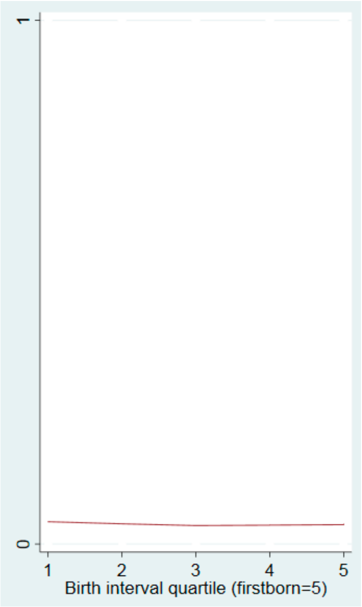

Figure S1. Plots of covariates with occurrence of breathing difficulties.
